# Evaluation of medical school faculty members’ educational performance in Korea in 2022 through analysis of the promotion regulations: a mixed methods study

**DOI:** 10.3352/jeehp.2023.20.7

**Published:** 2023-02-28

**Authors:** Hye Won Jang, Janghee Park

**Affiliations:** 1 Department of Medical Education, Sungkyunkwan University School of Medicine, Suwon, Korea; 2 Department of Medical Education, Soonchunhyang University College of Medicine, Cheonan, Korea; Hallym University, Korea

**Keywords:** Education, Medical school, Medical faculty, Republic of Korea

## Abstract

**Purpose:**

To ensure faculty members’ active participation in education in response to growing demand, medical schools should clearly describe educational activities in their promotion regulations. This study analyzed the status of how medical education activities are evaluated in promotion regulations in 2022, in Korea.

**Methods:**

Data were collected from promotion regulations retrieved by searching the websites of 22 medical schools/universities in August 2022. To categorize educational activities and evaluation methods, the Association of American Medical Colleges framework for educational activities was utilized. Correlations between medical schools’ characteristics and the evaluation of medical educational activities were analyzed.

**Results:**

We defined 6 categories, including teaching, development of education products, education administration and service, scholarship in education, student affairs, and others, and 20 activities with 57 sub-activities. The average number of included activities was highest in the development of education products category and lowest in the scholarship in education category. The weight adjustment factors of medical educational activities were the characteristics of the target subjects and faculty members, the number of involved faculty members, and the difficulty of activities. Private medical schools tended to have more educational activities in the regulations than public medical schools. The greater the number of faculty members, the greater the number of educational activities in the education administration and service categories.

**Conclusion:**

Medical schools included various medical education activities and their evaluation methods in promotion regulations in Korea. This study provides basic data for improving the rewarding system for efforts of medical faculty members in education.

## Graphical abstract

[Fig f3-jeehp-20-07]

## Introduction

### Background

The medical educational activities carried out by medical school faculty members are rapidly changing quantitatively and qualitatively. The recent shift of the medical education paradigm to competency-based medical education has required medical schools to organize and operate an educational system that enables individual students to successfully demonstrate their achievement of learning outcomes [1]. Therefore, the role of faculty members has expanded from traditional instructors to curriculum designers, mentors, advisors, and coaches so that learners can demonstrate the intended outcomes throughout the entire curriculum [2,3]. In addition, coronavirus disease 2019 challenged faculty members and students to an online learning environment, which led to a change from in-person education to online or hybrid education, and from large-scale lectures to small-group or individual-based learning [4,5].

Medical school faculty members struggle to carry out multiple duties: research, clinical practice, education, and public service. Despite increasing demands for engagement in education, education still ranks lowest in priority among their duties. This is mainly due to the fact that evaluation of educational performance is less systematic and its standards are less demanding than the evaluation of research performance and clinical practice performance. In order to be promoted, medical faculty members spend more time caring for patients and producing research results, rather than participating in education [6-8].

Efforts are being made to analyze the medical education activities of medical faculty members and to reward them appropriately for their dedication in education. The *Association of American Medical Colleges* (AAMC) developed a metrics system to measure faculty members’ effort and contributions to medical education and presented a list of educational activities that faculty members could conduct. Additionally, they proposed the concept of the relative value unit, which refers to the value of the weight of the unit of activities relative to the mission or policy of the medical school, to measure educational activities [9].

In Korea, a study has measured the efforts and contribution of medical faculty members in education at a single medical school [10]. The evaluation items and weights vary depending on the medical school’s mission; however, the items for fundamental medical education activities might be similar. Therefore, it is necessary to investigate the common evaluation items and weighting methods of medical education activities.

### Objectives

The aim of this study was to investigate the current status of evaluation of educational performance of medical faculty members through an analysis of promotion regulations of medical schools in Korea. Specifically, promotion regulations of each medical school were classified through content analysis, and contents of individual medical school’s promotion regulations were reorganized according to the classification.

## Methods

### Ethics statement

Ethical approval was not required for this study. This study did not include a clinical trial and did not collect any personal data. Information that was publicly available on websites was collected and analyzed.

### Study design

**Fig. 1.**
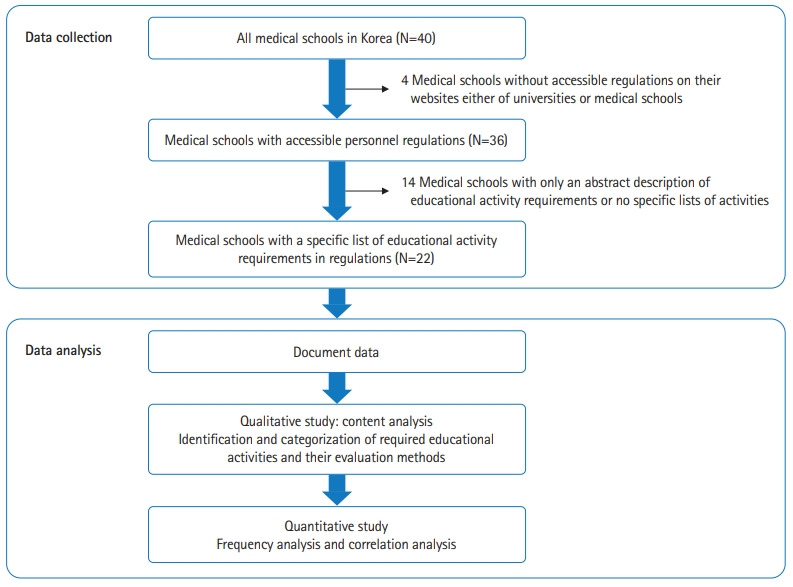
Data collection and analysis process.

This study used mixed methods. The strategy of this study was type 1 mixed methods [11,12], as this study connected the results of a qualitative study (phase 1) with the result of a quantitative study (phase 2) (Fig. 1).

### Phase 1: Qualitative study

#### Data collection

In August 2022, we searched the websites of medical schools and their universities in Korea to identify publicly accessible promotion regulations with provisions on medical educational activities. Data were collected from the promotion regulations of 22 out of 40 medical schools (Fig. 1).

#### Personal characteristics of the research team

The research team consisted of experts on medical education. J.P., a PhD in educational measurement and evaluation, and H.W.J., an MD, PhD in medicine, were professors in the Department of Medical Education at a school of medicine with several years of experience in the development of the faculty member evaluation systems.

#### Data analysis

Data were analyzed through deductive content analysis. This study defined and categorized the educational activities of faculty members recognized in the promotion regulations of each medical school according to the categories suggested in the AAMC framework to improve the correspondent validity and compared the results between the AAMC and our results [9]. The AAMC framework defines 4 educational activity categories: teaching (T), development of education products (D), education administration and service (A), and scholarship in education (S), and 17 main activities and 69 sub-activities. We added the student affairs (SA) category because many medical schools in Korea recognized work related to student affairs, such as student counseling and career coaching, as educational activities. Educational activities that did not fit into any of these 5 categories were classified as “others.” Two researchers (J.P. and H.W.J.) iteratively read and reviewed the contents in the collected promotion regulations, and codes were assigned to all documents. according to these 6 categories and their sub-activities. We also explored scoring methods of educational activities and factors for adjusting the weight of educational activities. The coding structure that emerged from the data was critically discussed between the researchers until consensus was achieved.

### Phase 2: Quantitative study

#### Variables

The coverage rate of educational activities in each category and the inclusion rate of individual educational activities in promotion regulations were analyzed. The type of institution, the year of establishment, and the number of faculty members at each medical school were variables for the basic characteristics of medical schools.

#### Data sources/measurement

Main data were the contents of individual medical school’s promotion regulations which were reorganized according to the classification based on the results of the qualitative research (Dataset 1). Data on medical schools’ basic characteristics were collected from the database of the Korean Association of Medical Colleges. Scoring methods and quality evaluation of medical educational activities is available at Dataset 2.

#### Bias

No selection bias was identified. This study included all medical schools that met the study’s eligibility criteria.

#### Study size

There are a total of 40 medical schools in Korea, and a survey was conducted on all schools, of which 22 met the research conditions. Therefore, no sample size was estimated for quantitative analysis.

#### Statistical methods

The quantitative variables were presented as frequency and percentage. Frequency, descriptive, and correlation analyses were performed. Correlations between the characteristics of medical schools and categories of educational activities were assessed using Spearman’s or Pearson’s correlation analysis. All data were processed in IBM SPSS ver. 27.0 (IBM Corp.).

## Results

### General characteristics of medical schools

Sixteen were private medical schools and six were public medical schools. Eight medical schools had 101–200 faculty members, seven had 201–300 faculty members, and seven had more than 300 faculty members. Ten medical schools were established after 1980, and the remaining 12 were established before 1980 (Table 1).

### Educational activities specified in the promotion regulations of Korean medical schools

Classifying medical educational activities stipulated in the promotion regulation of medical schools, the researchers yielded 6 categories and 20 activities with 57 sub-activities; teaching (5 activities and 16 sub-activities), development of education products (3 activities and 9 sub-activities), education administration and service (4 activities and 16 sub-activities), scholarship in education (5 activities and 12 sub-activities), student affairs (3 activities and 4 sub-activities), and others (Table 2). The “others” category had unique activities, such as donations, fundraising, medical students’ pass rate on the Korean Medical Licensing Examination, the pass rate on the specialty examination, disciplinary results, and so forth.

The coverage rate of educational activities in each category was the highest in the development of education products category (2.4/3, 78.8%). The second highest was teaching category (3.6/5, 71.8%), and the lowest was scholarship in education category (0.8/5, 16.4%) (Table 2). The inclusion rate of individual educational activities in promotion regulations was the highest in lecture activities, development of personnel, and direction of education components (21/22. 95.5%) (Table 2).

### Correlation and comparison between categories of educational activities and characteristics of medical schools

**Fig. 2.**
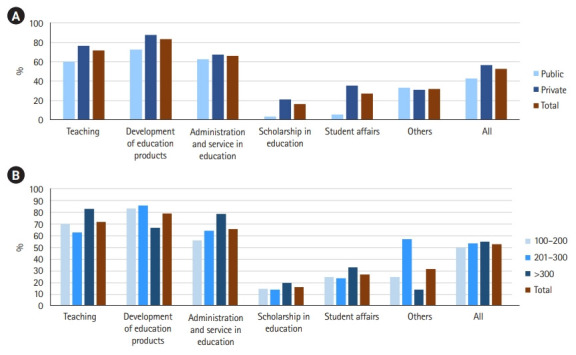
Comparison of the average number of the educational activities included in each category according to the type of medical schools (A) and the number of faculty (B). Development: development of education products. Administration: education administration and service, scholarship.

Private schools had a strong positive correlation with a longer history and the inclusion of medical educational activities in the promotion regulation in the following categories: teaching, scholarship in education, and student affairs. Private medical schools tended to include more educational activities than public medical schools (Fig. 2A, Supplement 1), and this tendency was prominent for scholarship and student affairs. The number of activities in the education administration and service category increased as the number of faculty members increased (Fig. 2B). The “scholarship in education” and “student affairs” had a moderate correlation with the “established year of school.” The correlation between individual activities and characteristics of medical school were shown in Table 3 [13] (Supplement 2).

### Evaluation of educational activities

#### Adjustment factors for the weight of educational activities

Four factors were identified to adjust the weight of medical educational activities; the characteristics of the target subjects, the characteristics of the faculty members, the number of faculty members involved in the activity, and the difficulty of the activity. Each factor, especially the difficulty of the activity, had various weighting methods. Fourteen medical educational activities were evaluated with more than one of these 4 weighting factors. The faculty members number was considered in evaluating lecture activities, small-group activities, and development of educational materials. The faculty members’ characteristics were considered only in evaluating the development of personnel (Table 4).

#### The scoring method and the quality evaluation

Most medical schools chose a criterion-referenced evaluation, with a scoring method based on raw data or grade scores within the range. For 14 medical educational activities, several medical schools displayed an upper limit on the points that could be received. There were 2 types of penalties: receiving negative points and rejecting performance (conditional approval). Examples of point deduction included failure to submit a lecture syllabus before the lecture, and the examples of rejection of performance included failure to meet the attendance time or frequency, such as participation in faculty member development seminars and committees. There were 3 types of evidence utilized to evaluate the quality of educational performance. Quality evaluation was conducted for 7 educational activities, among which lecture activities were evaluated most frequently (Table 5).

## Discussion

### Key results

This study provided an overview of the current evaluation status of medical school faculty members’ educational performance by identifying medical education activities and their assessment methods along with weighting factors through an analysis using mixed methods in Korea. The inclusion in the promotions regulations and the weighting and scoring method for the specific activity differed from school to school, which implied that medical schools demanded various educator roles from their faculty members depending on their mission and policies.

### Interpretation

While almost all medical schools included lecture activities in their promotion regulations, laboratory activities and clinical practice activities were included in relatively a small number of medical schools. Considering that many faculty members spend a considerable amount of time on them, effort seems necessary to reflect them more actively in the promotion regulations. The high inclusion rate of the development of personnel in promotion regulations seemed to be related to the standard of the Korean accreditation of medical schools such as “at least 50% of full-time faculty members must participate in medical education training or education-related faculty member development programs for at least 3 hours a year” [14].

Activities in the scholarship in education category, except for presentations in education, were rarely included in the regulations. Besides publishing papers, there are many activities related to scholarship in education, which include writing education research reports, making abstract-based presentations at educational academic meetings, giving symposium lectures, and so on. Medical schools should strive to ensure that all educational activities of faculty members are included in the promotion regulations, so that faculty members dedicated to medical education can be properly recognized and evaluated for their effort [6-8].

Among 4 adjustment factors for the weight of educational activities, the difficulty of the activity was the most frequently considered, and the number of items in each factor varied among medical schools. The AAMC recommended implementing quantitative and qualitative weights differently in stage 1, and completing the weights based on the mission of the school in stage 2 [9]. When establishing standards for evaluating faculty members’ educational performance, the weight of educational activities must be determined by reflecting the mission and policy of medical schools so that faculty members can accept the evaluation standards and devote themselves to educational activities consistent with the direction of the mission and policy.

Most educational activities were evaluated with a scoring system in which the more faculty members did, the more points they got. The penalty, either point deduction or conditional approval, was given when the activities were not properly carried out, or when the additional tasks necessary to complete the activities were not met.

### Limitations/suggestions

First, the data of this study were based on the promotion regulations published on the websites of medical schools or universities. There was a possibility that some universities/medical schools did not disclose the details of promotion regulations on the web.

Second, the small number of study subjects made further statistics other than correlation analysis impossible. In addition, caution may be required when interpreting the result of correlation analysis with a small sample number. Therefore, it would be appropriate to conduct a more comprehensive study using surveys and in-depth interviews through representative organizations such as the Korea Association of Medical Colleges.

### Conclusion

Rapidly changing medical education calls for more efforts of faculty members to meet the needs of learners and society. In order for faculty members to devote themselves actively and passionately to education, they must receive reasonable rewards. This study analyzed the status of medical education activities reflected in the promotion regulations of medical schools in 2022 in Korea. This study provides essential data for improving the reward system for medical faculty members’ educational efforts.

## Figures and Tables

**Figure f3-jeehp-20-07:**
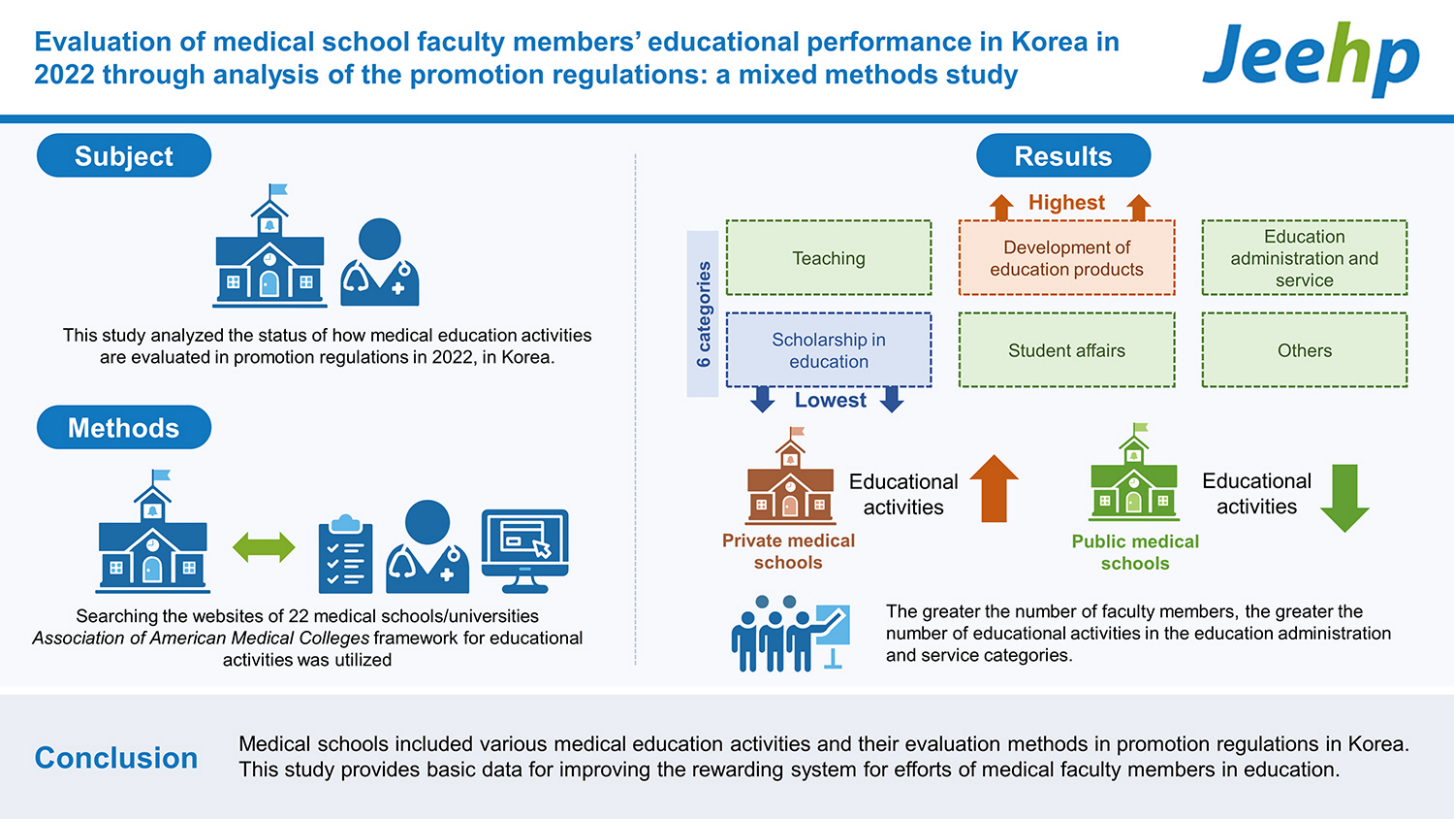


**Table 1.** General characteristics of the 22 medical schools

Type of medical school	Public	Private	Total
No. of faculty members			
101–200	1	7	8
201–300	3	4	7
>300	2	5	7
Year of establishment			
<1970	5	3	8
1970s	0	4	4
≥1980	1	9	10
Total	6	16	22

**Table 2.** List of medical educational activities and their inclusion rate specified in the promotion regulations of Korean medical schools

Category	Average no. of including activity	Educational activity	Inclusion rate % (no.)	Educational sub-activities	Examples
Teaching	3.6/5 (71.8)	T1. Lecture activities	95.5 (21/22)	● Lecturing in preclinical, clinical, or graduate courses	● Lecturing in a pre-med course
				● Lecturing during grand rounds	● Lecturing in a selective course
					● Lecturing in an integrative course
					● Lecturing in an English class
		T2. Laboratory activities	40.9 (9/22)	● Providing instruction in wet laboratory, computer laboratory, or skills laboratory activities	● Instruction in a wet laboratory
				● Providing instruction in research laboratory work	● Instruction in clinical skill lab (ICM)
		T3. Small-group activities (non-clinical)	77.3 (17/22)	● Serving as a tutor or facilitator in problem-based learning	● Tutor in problem-based learning
				● Serving as a small-group leader in a course	● Facilitator in team-based learning
				● Serving as a seminar leader	● Moderator/commentator of small-group discussion
					● Leading a seminar for graduate students
		T4. Individual activities (non-clinical)	90.9 (20/22)	● Serving as an individual tutor	● Mentor for academic underachievers
				● Serving as an advisor or mentor for students and trainees	● Research preceptor of medical students’ scholarly project
				● Serving as a research preceptor or thesis director	● Advisor of master’s degree students
				● Giving assistance with grant or manuscript preparation	● Member of doctoral review committee
		T5. Clinical activities	54.5 (12/22)	● Performing inpatient teaching during attending rounds	● Teaching during a clinical clerkship
				● Teaching in surgery or special clinical procedure rooms	● Conducting bedside teaching
				● Serving as preceptor for the student–house-staff patient care team	● Teaching in surgery rooms
				● Serving as an outpatient clinic attending	● Teaching in outpatient clinic
				● Serving as a case-based session leader on wards or in clinic	● Managing the students’ schedule
					● Participating in a mini-OSCE
					● Leading a case-based discussion
Development of educational products	2.4/3 (78.8)	D1. Development of educational units	63.6 (14/22)	● Developing a major curricular unit (e.g., course, clerkship, or laboratory program)	● Participating in major revision of the course
				● Developing a minor curricular unit (e.g., lab session, problem-based learning case, or conference)	● Developing a PBL module
				● Participating in a major revision of course, clerkship, laboratory, or other units	● Developing a CPX module, standardized patient scenario, checklist
		D2. Development of educational materials	77.2 (17/22)	● Developing innovative teaching methods, learning tools, or distance learning	● Developing the e-learning content
				● Developing a syllabus or manual (e.g., course or laboratory)	● Developing the test items (including KMLE, consortium test)
				● Developing teaching materials	
				● Developing examinations and other evaluation tools	
		D3. Development of personnel	95.5 (21/22)	● Participating in standardized patient orientation and training	● Participating in an internal/external faculty program
				● Developing faculty members and staff skills	● Participating in a workshop for newly recruited faculty members
Education administration and service	2.6//4 (65.9)	A1. Direction of educational components	95.5 (21/22)	● Serving as a program director (e.g., directing graduate or residency program)	● Director of graduate common course
				● Serving as a course director	● Course/clerkship director
				● Serving as a clerkship director	● Elective director (e.g., research, preclinical, clinical)
				● Serving as a laboratory director	● Director of review activities for certification examinations
				● Serving as an elective director (e.g., research, preclinical, clinical)	● Advisor/mentor of a club/grade
				● Serving as a director of review activities for certification examinations	
				● Serving as a student affairs director	
		A2. Evaluation of education	68.2 (15/22)	● Evaluating student, resident, or other trainee performance	● Evaluator of CPX, KMLE, portfolios
				● Evaluating major curriculum change	● Examination supervisor
				● Evaluating education programs	● Writing a self-evaluation report of the medical school
					● Participating in preparation of medical school accreditation
		A3. Administration of education	59.1 (13/22)	● Providing leadership at school level (e.g., education dean)	● Vice dean (education, research, planning)
				● Managing course, clerkship, laboratory, conference, or elective activities	● Head of an office/department of medical education
				● Designing and administering training programs, including research training	● Head of a research center
				● Contributing to facilities development and scheduling	● Chairman or member of an educational committee
				● Providing education committee service and leadership	
		A4. Special services	40.9 (9/22)	● Serving in outreach programs (e.g., K–12, college, community, and government)	● Teaching other medical professionals, the public, and residents
					● Participating in K-MOOC, open campus for high school students
Scholarship in education	0.8/5 (16.4)	S1. Research in education	9.1 (2/22)	● Submitting an education grant proposal (internal or external)	● Contribution of an educational project
				● Directing educational research or scholarly project (internal or external)	● Submitting a curriculum research report
		S2. Presentations in education	50.0 (11/22)	● Making internal presentations	● Presenter at an internal/external faculty members development program (seminar, workshop, etc.)
				● Making external keynote, plenary, or symposium lectures or presentations	● Presenter at an academic annual meeting
				● Making external abstract-based oral or poster presentations	
		S3. Service on editorial boards, review bodies, or in elected positions	13.6 (3/22)	● Serving as a book or journal editor	● Chair of an editorial board
				● Serving as an editorial board member or chair	● Reviewing manuscripts
				● Reviewing manuscripts, media, etc.	● Reviewing grants
				● Reviewing grants	
				● Serving in an elected office in an educational organization	
				● Providing consultations in education	
		S4. Receiving education awards and prizes (internal and external)	9.1 (2/22)	● Receiving education awards and prizes (internal and external)	● Best teacher award
					● National/public institute/community educational award
Student affairs	1.2/3 (40.9)	SA1. Participating in admission	36.3 (8/22)	● Participating as an evaluator in the student admission examination	● Serving as evaluator in admission interview/entrance examination
				● developing admission examination items	● Developing admission interview item
		SA2. Counseling and mentoring	59.1 (13/22)	● Counseling or mentoring students	● Student counseling
					● Career counseling
		SA3. Supporting extracurricular activities	27.3 (6/22)	● Participating in extracurricular activities	● Participating in graduation trips, field training, orientations, retreats
Others	0.3/1 (31.8)	O1. Others	31.8 (7/22)	● Others	● Donation, fundraising, performance of clinical practice in hospital, pass rate of the Korean National Licensing Examination for Physicians, pass rate of specialty examinations, stages of disciplinary action

ICM, introduction to clinical medicine; OSCE, objective structured clinical examination; PBL, problem-based learning; CPX, clinical performance examination; KMLE, Korean Medical Licensing Examination; K-MOOC, Korean Massive Open Online Course.

**Table 3.** Correlation between categories of medical educational activities and characteristics of medical schools

	Medical school			Categories of medical educational activity					
	Public/private (1)	No. of faculty members (2)	Established year (3)	T	D	A	S	SA	O
No. of faculty members (2)	-0.11								
Established year (3)	0.52	-0.28							
Teaching (T)	0.31	0.15	0.19						
Development of educational products (D)	0.08	-0.19	0.12	-0.02					
Administration and service (A)	0.07	0.38	0.12	0.36	0.17				
Scholarship in education (S)	0.44	0.01	0.34	0.29	0.05	0.09			
Student affairs (SA)	0.44	0.01	0.34	0.29	0.05	0.09	1.00		
Others (O)	-0.02	-0.14	0.00	-0.34	0.26	-0.14	0.02	0.02	
Total	0.30	0.18	0.27	0.55	0.35	0.59	0.42	0.42	0.03

The correlation coefficient was interpreted as follows with a positive or negative direction; very weak correlation (<0.2), weak correlation (0.20–0.35), moderate correlation (0.35–0.50), strong correlation (0.50–0.70), and very strong (>0.70) [13].

**Table 4.** Factors for weight adjustment of medical educational activities

Educational activities	Characteristics of the subject	Characteristics of the faculty members	Faculty members number	Difficulty of the activity
T1. Lecture activities	● Undergraduate/graduate		● Single/team-teaching	● Language: lecture in English/Korean
				● Mode of learning: online/in-person
				● Number of students: large/medium/small
				● Lecture time
T3. Small-group activities (non-clinical)			● Single/multiple	● Types of activity: TBL/CBL/others
				● Number of students: less than 20/20 or more
T4. Individual activities (non-clinical)	● Undergraduate/MD/PhD			
	● Domestic/international			
T5. Clinical activities				● Type of teaching: bedside teaching/clinical skills training/teaching patient encounter
D1. Development of educational units				● Type of unit: PBL module/CPX module
D2. Development of educational materials	● Domestic/international		● Single/multiple	● Role: leader/participant
				● Development/edition or selection of items
D3. Development of personnel	● Newly recruited/general faculty members	● Clinical/basic science department		● Program hosting institute: internal/external
				● Domestic/international program
				● Program duration: half-day/full-day
A1. Direction of educational components	● Club/grade			● Role: director/vice-director/specialty director/subspecialty director
				● Type of the subject: major/liberal arts
				● Curricular phase: pre-medical/medical
				● Duration of clerkship: ≤10 weeks/>10 weeks
A2. Evaluation of education				● Type of examination: written exam/oral exam
				● Object of examination: knowledge/skills
				● Evaluation hosting: internal (medical school)/external (consortium)
				● Evaluation time
				● Contribution: participating in evaluation meetings/writing evaluation report
A3. Administration of education				● Position: vice dean/head of office of medical education
				● Role: chair/member
S1. Research in education				● Contribution to educational projects
S3. Service on editorial boards, review bodies, or in elected positions				● Role: chair/member
S4. Receiving education awards and prizes (internal and external)				● Awarding body: domestic/international
SA1. Participating in admission				● Type of admission: early/regular
				● Type of evaluation: interview/document evaluation

TBL, team-based learning; CBL, case-based learning; MD, Doctor of Medicine; PhD, Doctor of Philosophy; CPX, clinical performance examination.

**Table 5.** Scoring methods of medical educational activity

Category	Educational activity	Addition		Subtraction		Type of evidence for quality evaluation				
		Upper limits	Minimum requirement	Point deduction	Conditional approval	Student	Faculty members	Committee	Student+faculty members	Student+committee
Teaching	T1	14		7	2	5		1	4	1
	T2	4					1			
	T3	10		1	1					
	T4	13			1			1		
	T5	7		1		1	1			
Development of educational products	D1	9			3			1		
	D2	8	1	1	1					
	D3	12	2	1	1					
Education administration and service	A1	10			1			1		
	A2	6								
	A3	6			2		1	1		
	A4	6								
Scholarship in education	S1	1								
	S2	2								
Student affairs	SA1	2								
	SA2	13	2	1	4					
	SA3	2								
Others	O1			1	1					

T1, lecture activities; T2, Laboratory activities; T3, small-group activities (non-clinical); T4, individual activities (non-clinical); T5, clinical activities; D1, development of educational units; D2, development of educational materials; D3, development of personnel; A1, direction of educational components; A2, evaluation of education; A3. administration of education; A4, special services; S1, research in education; S2, presentations in education; S3, service on editorial boards, review bodies, or in elected positions; SA2, counseling; SA3, supporting extracurricular activity; O1, others.

## Footnotes

**Data availability**
Data files are available from Harvard Dataverse: https://doi.org/10.7910/DVN/FL823U Dataset 1. Educational activities in regulations.

Dataset 2. Scoring methods and quality evaluation of medical educational activities.

## Supplementary materials

Supplementary files are available from Harvard Dataverse: https://doi.org/10.7910/DVN/FL823U

Supplement 1. Data for presentation of Fig. 2.

Supplement 2. Correlation between individual activities and characteristics of medical school.

Supplement 3. Audio recording of the abstract.
